# 

**Published:** 1799-05

**Authors:** 


### NEW PUBLICATIONS IN APRIL.

*Medicina Nautica:* An Essay on the Diseases of Seamen; with communications by American Physicians on the new doctrine of Contagion and Yellow Fever. By Thomas Trotter, M.D. Vol. II. *London.* Price 7s. 6d. Longman and Rees.

Ten Minutes Advice to the Afflicted in Nervous Disorders, by a Student in Medicine. *London.* Price 6d. Rivingtons.

(*The new Foreign Publications in our next Number.*)

---

### TO CORRESPONDENTS.

WE think it a duty incumbent upon us to acknowledge the favour of many communications we have received from our friends, as well as anonymous correspondents: but while we mention this flattering encouragement with peculiar gratitude and satisfaction, we cannot help observing, that several papers signed merely with initial letters—in answer to others accompanied with the signatures of their authors—have been transmitted to us for insertion. As it would evince a degree of injustice to admit such articles, especially on *controversial subjects*, we hope, that this will be considered a sufficient apology for their not appearing.—And as the names of places and persons, if written in a slight and imperfect manner, give frequent occasion to mistakes and errors of the press, our correspondents are particularly requested to write their names, as well as designations, *distinctly*, and *without abbreviations*.

There is another circumstance which we cannot pass over in silence: it relates to communications sent by empirics, with a view either to extol their medicines, or recommend their pamphlets—both are inconsistent with our professions, and the duties we owe to the public. Indeed, it has not entered into *our* plan, to circulate the prospectus of this Journal among the proprietors and venders of Nostrums; hence we shall, upon this occasion, only observe: "*Quod* MEDICORUM *est, promittunt* MEDICI:"—*sed non omnes, qui* VERAM *promittunt*, VERAM MEDICINAM *colunt*.

Lastly, it being always desirable, that communications on *practical subjects* be laid before the public as soon as possible, we again request our correspondents to favour us with such articles *before the 15th of the month;* as otherwise, from our inability to insert them, both they and our readers must inevitably be disappointed.

### TO THE PUBLIC.

THE Proprietor of the MEDICAL AND PHYSICAL JOURNAL feels it his duty gratefully to acknowledge the liberal patronage with which the work has hitherto been honoured. It affords him, at the ſame time, the higheſt ſatisfaction to learn, that the plan and execution meet with the general approbation of the Faculty. The friends of the undertaking will doubtleſs be gratified to learn, that the ſale of the two firſt numbers has already conſiderably exceeded that of any Medical Journal hitherto publiſhed in this country.

The great variety of valuable and original communications, authenticated by the reſpectable ſignatures of the writers, evinces, in the moſt unequivocal and flattering manner, the approbation and preference of the moſt intelligent of the Faculty. Teſtimonials ſo honourable and ſatisfactory, place beyond a doubt the complete and permanent eſtabliſhment of the work.

The Proprietor cannot refrain, in juſtice to his own intereſt, and in confirmation of the preceding remarks, to ſubjoin copies of letters, with which he has been honoured from the three principal Medical Societies in the metropolis.

To Mr. PHILLIPS.

*SIR,*

*I am requeſted by the unanimous vote of the Phyſical Society, to return you their ſincere thanks for the firſt and ſecond numbers of the Medical Journal, which with much pleaſure they add to their library.*

*I remain, Sir,*

*Your very humble Servant,*

*Medical Theatre, Guy's Hoſpital,* GEORGE JOHNSON, *Secretary.*

*April* 13, 1799.

---

To Mr. PHILLIPS.

*SIR,*

*I am deſired by the Lyceum Medicum Londinenſe, to return their thanks to you, for preſenting them with the Medical and Phyſical Journal.*

*I am, Sir,*
*Your's, &c.*

*Lyceum Medicum Londinenſe,* J. CRUKE, *Regiſter.*

*April* 16, 1799.

---

To Mr. PHILLIPS.

*SIR,*

*The Preſidents and Members of this Society voted you their unanimous thanks on the 9th inſt. and ordered me to tranſmit the ſame by letter, for your liberal donation of the Medical and Phyſical Journal, which they had previouſly reſolved to purchaſe.*

*I am, Sir,*

*Your obedient Servant,*

CORNWALLIS HUNT, *Secretary.*
*Medical Society, St. Bartholomew's Hoſpital,*
*April* 25, 1799.

